# ChatGPT-4 and Human Researchers Are Equal in Writing Scientific Introduction Sections: A Blinded, Randomized, Non-inferiority Controlled Study

**DOI:** 10.7759/cureus.49019

**Published:** 2023-11-18

**Authors:** Binyamin Sikander, Jason J Baker, Can D Deveci, Lars Lund, Jacob Rosenberg

**Affiliations:** 1 Surgery, Herlev Hospital, Herlev, DNK; 2 Urology, Odense University Hospital, Odense, DNK

**Keywords:** natural language processing, chatbot, artificial intelligence and writing, artificial intelligence in medicine, gpt-4, chatgpt

## Abstract

Background

Natural language processing models are increasingly used in scientific research, and their ability to perform various tasks in the research process is rapidly advancing. This study aims to investigate whether Generative Pre-trained Transformer 4 (GPT-4) is equal to humans in writing introduction sections for scientific articles.

Methods

This randomized non-inferiority study was reported according to the Consolidated Standards of Reporting Trials for non-inferiority trials and artificial intelligence (AI) guidelines. GPT-4 was instructed to synthesize 18 introduction sections based on the aim of previously published studies, and these sections were compared to the human-written introductions already published in a medical journal. Eight blinded assessors randomly evaluated the introduction sections using 1-10 Likert scales.

Results

There was no significant difference between GPT-4 and human introductions regarding publishability and content quality. GPT-4 had one point significantly better scores in readability, which was considered a non-relevant difference. The majority of assessors (59%) preferred GPT-4, while 33% preferred human-written introductions. Based on Lix and Flesch-Kincaid scores, GPT-4 introductions were 10 and two points higher, respectively, indicating that the sentences were longer and had longer words.

Conclusion

GPT-4 was found to be equal to humans in writing introductions regarding publishability, readability, and content quality. The majority of assessors preferred GPT-4 introductions and less than half could determine which were written by GPT-4 or humans. These findings suggest that GPT-4 can be a useful tool for writing introduction sections, and further studies should evaluate its ability to write other parts of scientific articles.

## Introduction

Writing high-quality scientific articles is essential when communicating research findings to the scientific community. Introduction sections of scientific articles play an important role in providing the necessary context, identifying the research gap, and establishing the aim [1]. Artificial intelligence (AI) is rapidly advancing, leading to the development of AI systems that perform a wide range of tasks in the research process [2,3]. Specifically, natural language processing models such as Chat Generative Pre-trained Transformer (ChatGPT) and the newest model GPT-4, developed by OpenAI (San Francisco, CA) [4], have been utilized in various applications. These models mimic human-like conversations and provide appropriate responses. They are regularly enhanced with reinforcement techniques and machine learning to improve their understanding and responsiveness to users' inquiries. You can ask GPT-4 anything and receive human-like replies to your questions or requests [5]. This includes manuscript writing [6], and assessment for grammar, spelling, and style [7]. While there is a growing interest in using AI-synthesized text for academic purposes [8], AI-synthesized text could be a potential tool for researchers in the development of their articles [3]. We hypothesized that GPT-4 could write scientific introduction sections just as well as human researchers regarding publishability, readability, and content quality.

This study investigated whether GPT-4 and humans were equal in writing scientific introduction sections in a randomized blinded non-inferiority design.

## Materials and methods

This blinded, randomized, non-inferiority study was reported according to the Consolidated Standards of Reporting Trials extension for non-inferiority and equivalence randomized trials (CONSORT non-inferiority) and artificial intelligence (CONSORT-AI) [9,10]. The study was conducted within our research network, and no changes were made to the method after the study’s commencement. The 18 latest articles published in "The Lancet eClinicalMedicine," an open-access journal that covers all medical specialties, were selected to have their introduction sections reproduced using GPT-4. In the pilot phase, we found that using one command for all study types was ineffective, so we limited our analysis to the 18 latest comparative original studies. The study consisted of two parallel groups: one group with human-written introductions from already published articles and another group with introductions synthesized by GPT-4. Both introduction sections had the same aim of study. The 18 pairs of introduction sections were distributed amongst eight blinded assessors, each receiving six to seven pairs of introductions in a random sequence, ensuring that each pair would be evaluated three times. The blinded assessors who judged the introductions were either novel- or non-users of ChatGPT and were required to have both a Doctorate of Medicine (DM) and Doctor of Philosophy (Ph.D.) and published at least four articles as first author, and they were recruited by convenience sampling in our research network. The GPT-4-synthesized introductions comprised of background, research gap, and aim for a scientific journal. The GPT-4 was fed with the aim from the already published original article and was instructed to “Write an introduction section including background, research gap, and aim for a scientific journal with the aim ‘Insert aim’” followed by a request to “make the background more detailed.” The GPT-4-synthesized introduction sections were altered in text size and font to match the originals, but no changes were made to language or words (Supplementary File 1). We removed references from the original articles as GPT-4's output does not automatically include references, and also since this was beyond the scope of this study.

The primary outcome was to compare the GPT-4-synthesized with human-written introduction sections regarding publishability, readability, and content quality using 1-10 Likert scales. A questionnaire was developed for the blinded assessors to evaluate the introduction sections (Supplementary Files 2-4). To ensure the questionnaire's design, several pilot tests were conducted. Additionally, the questionnaire was face-validated with persons who potentially could be included as assessors (but not participating in the study) to ensure the understanding and interpretation of each question [11]. After reviewing the comments provided following face-validation, the author group made minor adjustments to the questionnaire. A score of 10 on the Likert scale indicated that no changes were needed for publishability, readability, or adequate detail for content quality, while a score of 1 signified unpublishable or unreadable content or improper detail level. For content quality scores below 10, additional questions were asked to identify whether there was a lack of details, excessive details, or a combination of both.

The secondary outcome involved an assessment of the introduction’s sections, whereby assessors were asked to indicate their preferences and identify whether the introductions were generated by GPT-4 or humans, providing their reasoning in free text. The free-text answers were grouped into similar answer options. An objective language analysis was conducted to compare the readability of GPT-4-synthesized and human-written introductions. This was done using online calculators for Lix [12,13] and Flesch-Kinkaid [14,15] scoring systems for readability evaluating factors such as sentence length, number of words, and number of letters per word.

Sample size

The sample size calculation was based on continuous data from a Likert scale ranging from one to 10, using an online calculator [16]. The least relevant difference was set to one point on the Likert scale. The calculation was based on the formula n = f (α, β) × 2 × σ2 /d2, where the significance level (alpha) was set at 5%, the statistical power (1-beta) at 90%, the standard deviation of the outcome (σ) was set as 1, and the non-inferiority limit (d) was set at 1. This indicates that GPT-4-synthesized introductions could be considered non-inferior if they were ≤1 point worse or better. This resulted in a sample size of 18 samples per group.

Randomization and blinding

An online tool [17] was used to generate a randomized allocation sequence for the distribution and sequence of the introduction section pairs to the blinded assessors. This ensured variation in the order of exposure of GPT-4 and human-written introduction sections. Each pair of introduction sections was numbered from one to 18 and assigned as either number 1 or 2 for allocation concealment. Only the first author knew the order of randomization.

Statistical analyses

Statistical analyses were conducted with IBM SPSS Statistics for Windows (version 28.0., IBM Corp., Armonk, NY), and graphic illustrations with Microsoft Excel for Windows 2023 (version 16.72, Microsoft Corporation, Redmond, WA). Normal distribution was assessed with Shapiro-Wilk tests, histograms, and Q-Q plots. Since most continuous data were non-normally distributed, all data were analyzed with the Mann-Whitney U test to uniformly present data. Descriptive statistics for continuous data were presented as median and range. Categorical data were presented as crude rates and compared with the chi-squared test. In the non-inferiority design, a difference of ≤1 point in the Likert scale was considered as not relevant regarding publishability, readability, and content quality. Since each introduction section was independently assessed by three assessors, we used the Fleiss' kappa method to determine the inter-rater agreements and thereby investigate the consistency of the assessments. Fleiss' kappa was chosen because it accounts for chance and does not assume that the same assessors rated all items [18]. According to the Landis and Koch scale, we classified inter-rater agreement as follows: very good (>0.8-1.0), good (>0.6-0.8), moderate (>0.4-0.6), fair (0.2-0.4), and poor (<0.2) [19].

Ethics

According to the Danish legislation, approval from the ethics committee was not required [20]. Additionally, Danish Data Protection Agency approval was not required as personal data were not processed in the project.

## Results

Table 1 presents the results of the assessment of GPT-4-synthesized and human-written introduction sections by the blinded assessors. It shows the median scores and ranges for publishability, readability, and content quality. The 18 pairs of introduction sections were assessed three times, resulting in a total of 54 assessments. There was no significant difference between GPT-4 and human introductions in terms of publishability and content quality. However, GPT-4 had a significantly higher score on readability, but the difference was less than one on the Likert scale, which was considered a non-relevant difference.

**Table 1:** Summary of assessment The eight blinded participants' assessment of 18 Generative Pre-trained Transformer 4 (GPT-4) synthesized vs. 18 human-written published introductions. Each introduction section was assessed three times, resulting in an assessment of 54 (3 x 18) introduction sections in each group. The level of detail for content quality is presented in quotation marks. n: number; %: percentages. The table shows the average and range for Likert scales, reflecting the collective opinions of respondents. A higher average indicates stronger agreement. The Lix score measures text complexity based on sentence length, while the Flesch-Kincaid score assesses readability based on word and sentence length. Lower scores suggest easier readability.

Assessment			GPT-4	Humans	P-value
Evaluation criteria					
	Publishability, median (range)		9.0 (3–10]	8.5 (1–10)	0.061
	Readability, median (range)		9.0 (2–10]	8.5 (1–10)	0.010
	Content quality, median (range)		9.0 (2–10]	9.0 (1–10)	0.119
		“Too superficial,” n (%)	13 (24)	1 (2)	
		“Too detailed,” n (%)	9 (17)	21 (39)	
		“Lacking/too detailed in some areas,” n (%)	11 (20)	11 (20)	
		“No need for change,” n (%)	21 (39)	21 (39)	
Readability test					
	Flesch-Kincaid, median (range)		20 (16–22)	18 (14–21)	0.013
	Lix score, median (range)		72 (61–76)	62 (53–78)	0.002

**Figure 1:**
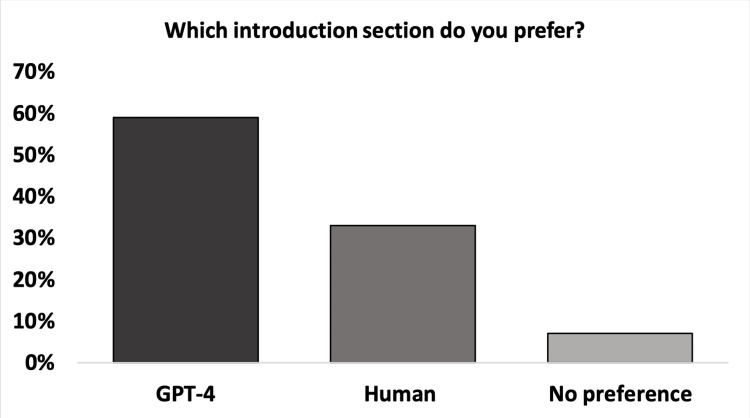
The blinded assessors’ preferences of either the Generative Pre-trained Transformer 4 (GPT-4) or human-written introductions The difference between the assessment for GPT-4 and human introductions was significant (p < 0.02).

Most of the assessors preferred the GPT-4 introductions (59%, n = 32), while 33% (n = 18) preferred the human-written introductions, and 8% (n = 4) had no preference (Figure 1). This difference in preference between GPT-4 and human introductions was significant, meaning there was a clear distinction in people's preferences (*P < 0.02). Among those who preferred GPT-4 introductions, the free-text comments revealed that 50% (n = 16) chose them because of "better flow and formulation," 41% (n = 13) preferred them for being "short and concise," and 9% (n = 3) believed that they better expressed "details according to the aim." When the human-written introductions were preferred, 72% (n = 13) reported preferring them due to "more relevant background information and GPT-4 being too superficial," 17% (n = 3) reported "better flow and formulation," and the remaining 11% (n = 2) preferred them due to "better structure."

**Figure 2:**
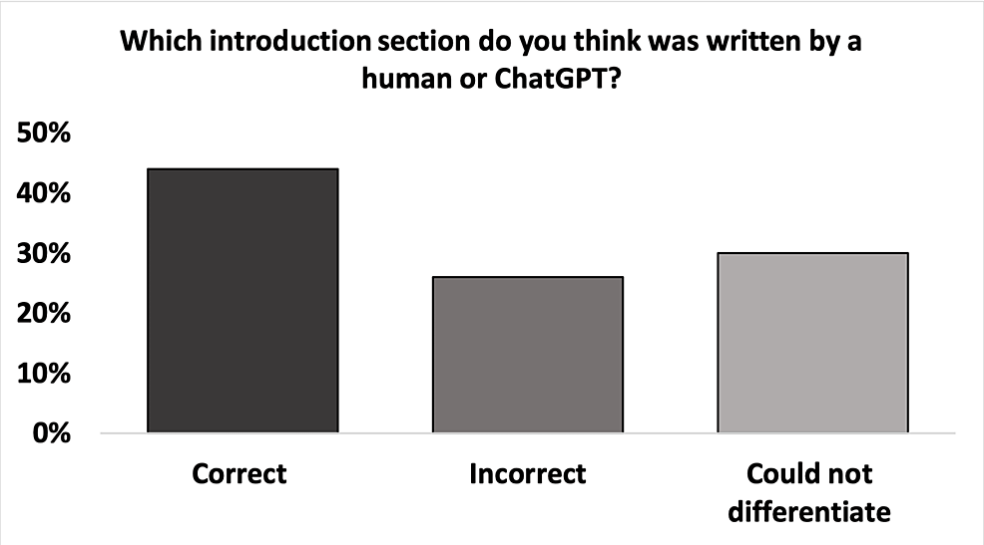
The blinded assessors’ guess on whether the introduction section was Generative Pre-trained Transformer 4 (GPT-4) or human-written The difference between the assessment for GPT-4 and human introductions was significant (p < 0.02).

Figure 2 presents the assessors' guesses of whether they thought the introduction sections were written by GPT-4 or humans. A total of 44% (n = 24) guessed correctly, and the remaining 56% (n = 30) could either not differentiate or assumed incorrectly. Among those who guessed correctly, 63% (n = 15) stated that GPT-4 introductions were “too superficial and less factual” compared with the human-written introductions, while 37% (n = 9) stated that the “formality in the language and generic textbook introduction structure” could tell the two apart. Of those who guessed correctly, there was an equal 50% (n = 12) distribution between the preference for GPT-4 and human-written introductions.

The calculated Fleiss' kappa for all three parameters expressed poor inter-rater agreement amongst the three blinded assessors. The kappa coefficients and P-values were as follows: (k) = 0.059 (CI 95%: -0.34 to 0.152) and P = 0.212 for publishability; (k) = -0.007 (CI 95%: -0.098 to 0.084) and P = 0.887 for readability; and (k) = 0.081 (CI 95%: -0.008 to 0.170) and *P = 0.073 for content quality.

According to Flesch-Kincaid's grade levels and Lix scores (Table 1), the introduction sections synthesized by GPT-4 had significantly higher scores for readability, indicating that they should have been harder to read compared with the human-written introductions. However, in the Flesch-Kincaid grading level, both human-written and GPT-4-synthesized introductions were assessed as “college graduate level” in terms of difficulty. The Lix scores also indicated that they were in the same category “Very hard” in terms of difficulty. The difference between the GPT-4-synthesized and human-written introductions was two points on the Flesch-Kincaid grade level and 10 points on the Lix score level, which represents a 10% difference on both scales [12,14].

## Discussion

This study found that GPT-4-synthesized introductions were equally as good as human-written introductions, with no significant differences in terms of publishability and content quality. While GPT-4 had a significantly better readability score, this small difference was not considered relevant. Furthermore, the introduction sections created by GPT-4 were frequently preferred over the human-written introductions. Less than half of the assessors correctly guessed which introduction sections were written by GPT-4 or humans. The remaining majority was distributed almost evenly among those who were not able to differentiate or guessed incorrectly. Finally, when comparing the introduction sections with the Flesch-Kincaid and Lix scores, the readability scores for GPT-4-synthesized introductions were significantly higher than those for human-written introduction sections. The objective readability tests showed that text written by GPT-4 had longer sentences and words. We could have told GPT-4 to make them shorter, but the assessors found the GPT-4 written text easier to read, indicating that it was unnecessary.

GPT-4 is a useful tool for various research writing tasks, including generating drafts for research protocols, manuscripts, grant proposals, and patient education materials [21]. In the grouped responses for the preference for human introductions, a proportion reported that the information provided by GPT-4 was “too superficial.” However, a matching proportion of the grouped responses that preferred the GPT-4 introductions reported that the content was short and well-connected to the aim. This leaves the decision inconclusive as to whether GPT-4 can produce content that would saturate the needs of a scientific article. Nonetheless, we believe that GPT-4 should be seen as an assisting tool to improve the overall quality of the content produced by researchers. Furthermore, GPT-4 can improve the quality of existing text and eliminate errors [21,22]. This is supported by our findings that the majority of assessors preferred the GPT-4-synthesized introductions over the human-written introductions. Approximately 50% of the reasoning for GPT-4 being preferred over human-written introductions was due to “better flow and formulation” in terms of the writing.

No previous study has compared the readability scores between GPT-4 and human-written text through objective means of scoring (Flesch-Kincaid and Lix). However, in the subjective assessment of readability, GPT-4 scored higher than human-written introductions across the 18 introduction pairs for comparison. The median difference between GPT-4 and human-written text was both relevant and statistically significant for both scoring tools as the 10% difference indicates a different grading of readability. The major reason for preferring the GPT-4-synthesized introductions over the human-written ones was due to “better flow and formulation” referring to better readability of the text. These differences between the subjective and objective assessment may be due to how the scoring tools calculate readability, based on the length of sentences and words providing a grade or score that indicates objective readability [12,14]. However, they might not be usable in this context as they do not consider the flow and formulation of language.

The strengths of this randomized and blinded non-inferiority study include reporting according to CONSORT non-inferiority and AI guidelines [9,10] and the validation of the questionnaire [11]. The inter-rater agreement was poor, but the bias from the subjective assessment was reduced because each pair of introduction sections was reviewed by three different assessors, thus increasing the reliability of the assessments. We conducted subjective and objective readability assessments to get a comprehensive overview of the GPT-4 and human introduction sections. The assessors had a minimum of a Ph.D. degree to ensure a suitable research background. This study also had some limitations such as the setting of the standard deviation at 1, which could potentially underestimate the true variability and complexity of the data. The inclusion of introduction sections from a single journal focused on comparative studies may limit generalizability. However, the journal from which the introduction sections were acquired covers a broad variety of medical specialties, such as cardiology, neurology, immunology, psychiatry, and gastroenterology, which enhances generalizability. Furthermore, the command given to GPT-4 was simple and based on the general format for introductions and the aim of the originally published articles. Assessors were recruited through convenience sampling, ensuring no interaction between participants and blinding the distribution of introduction sections. Previous concerns regarding GPT-4's association with the research process stem from the lack of validity in its data output [22].

AI is increasingly integrated into our daily lives, expanding its presence in both the researcher [22,23] and clinicians' daily workflow [24,25]. Our study revealed that researchers with a Ph.D. degree or higher rated GPT-4 introductions as non-inferior to human-written published introduction sections. GPT-4 will undoubtedly impact various stages of the research process in the near future, and maintaining transparency in academic research to uphold ethical standards is therefore a priority [3]. GPT-4 is applicable in assisting researchers in crosschecking introduction sections for suggestions to improve readability without affecting content quality or publishability [21,22]. Thus, it has the opportunity to increase the effectiveness and time consumption in the writing process of scientific articles. Future research should focus on the potential of AI and related risks to incorporate GPT-4 and other AI models in the research process. Future studies could also investigate the use of AI tools to assist in other areas of the scientific research process.

## Conclusions

In conclusion, GPT-4 was rated as at least equal to humans in writing introduction sections in terms of publishability, readability, and content quality. Most of the assessors preferred the GPT-4 introductions due to their better flow and formulation, being short and concise, and presenting details according to the aim of the studies. In contrast, human-written introductions were preferred by some, due to more relevant background information and better structure. The Lix and Flesch-Kincaid scores were significantly higher for introductions written by GPT-4, indicating that they should have been more difficult to read. However, this was not the case in the subjective assessment of readability, probably due to better flow and structure. The findings in this study suggest that GPT-4 can be a valuable tool in writing introduction sections, but the use would require research-based background knowledge to ensure the validity of the output.

## Figures and Tables

**Figure 3:**
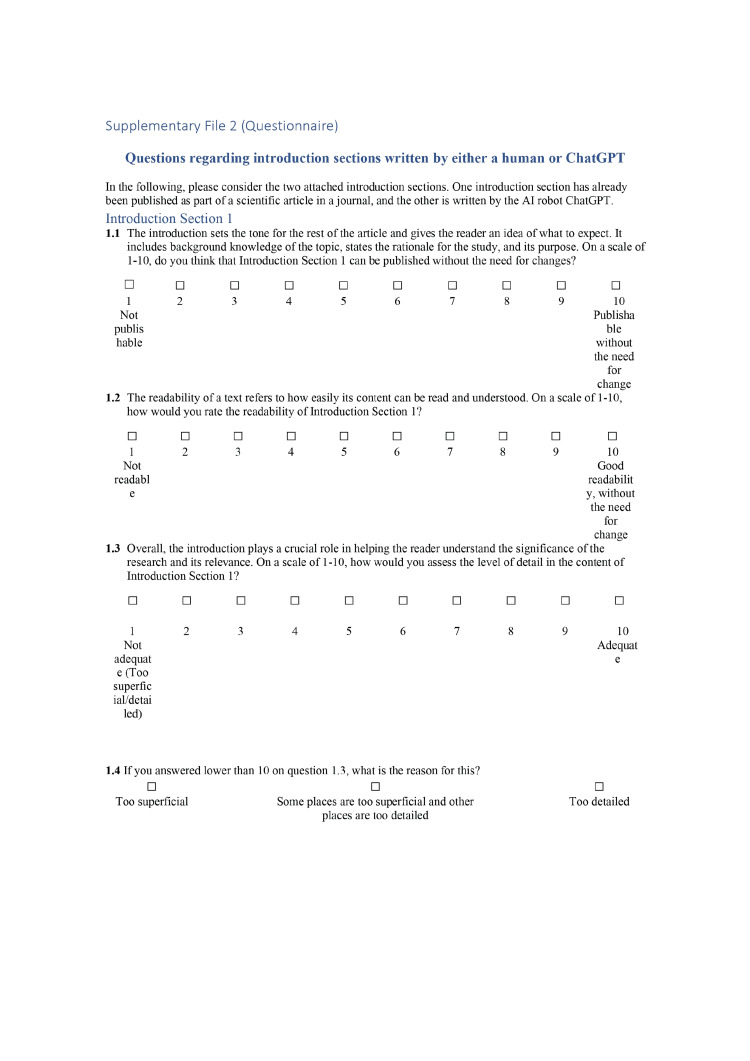
Questions regarding introduction sections written by either a human or ChatGPT

**Figure 4:**
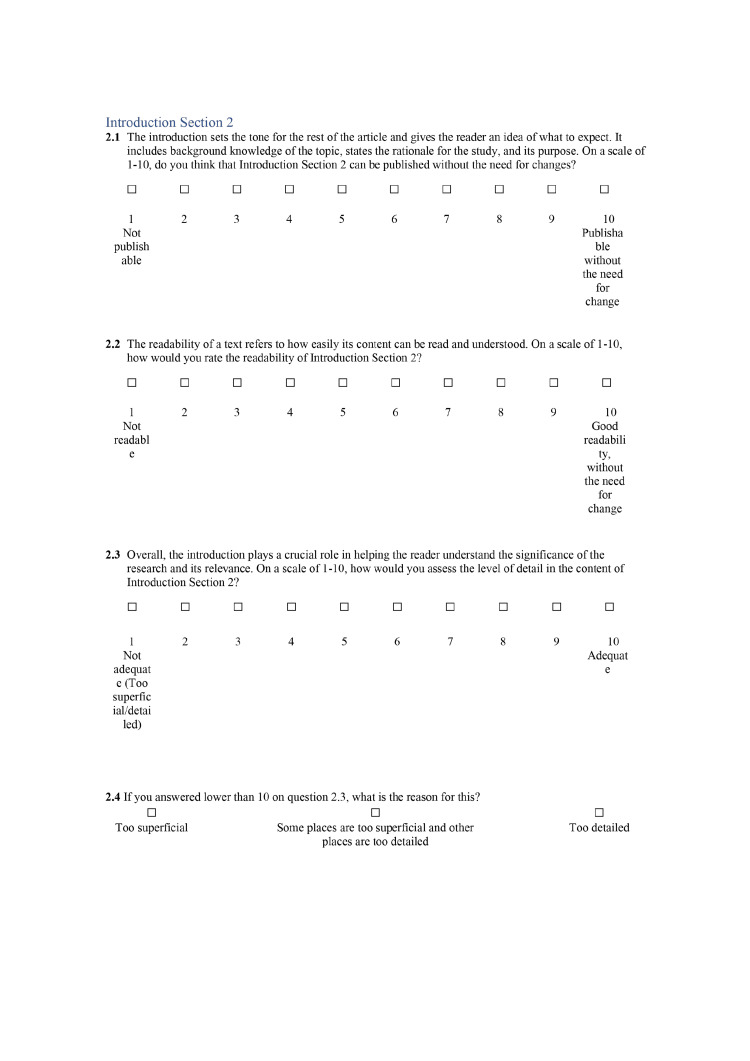
Questions regarding introduction sections written by either a human or ChatGPT

**Figure 5:**
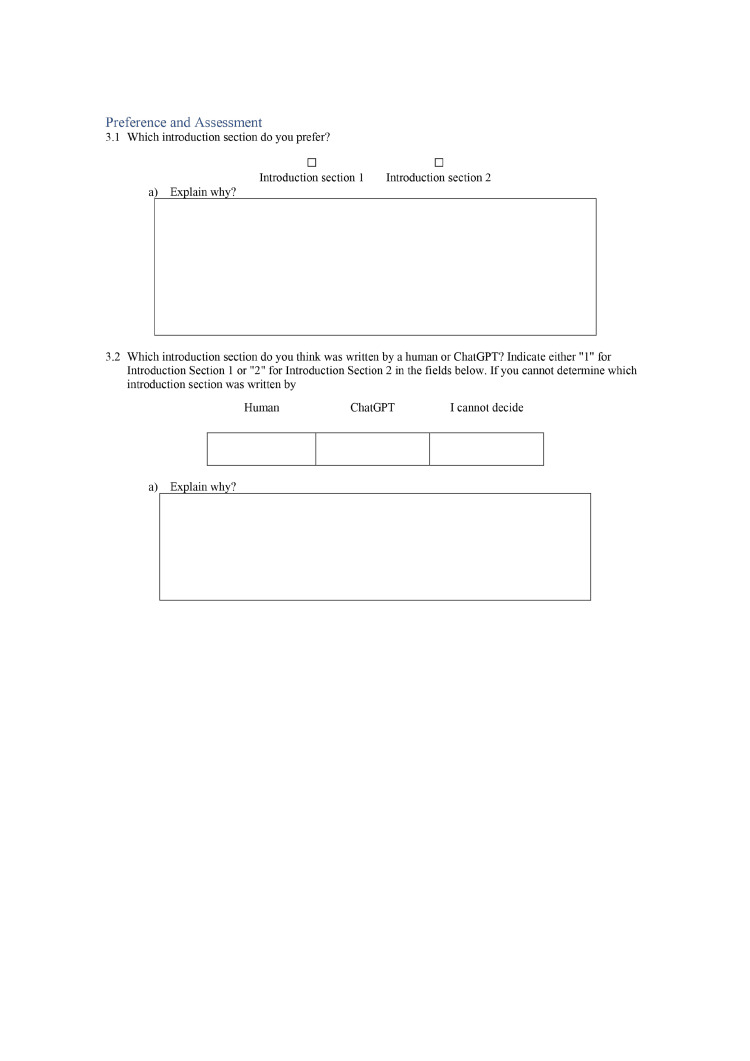
Questions regarding introduction sections written by either a human or ChatGPT Preference and assessment.

## Appendices

 Supplementary file 1

**Table 2:** Example of prompts for creating a scientific introduction section given to GPT-4

Prompt:* "Write an introduction section including background, research gap, and aim for a scientific journal with the aim."* Answer: The study aimed to analyze kidney function trends before and after COVID-19-related AKI in a large, hospitalized cohort and to identify factors linked to mortality and impaired long-term kidney recovery. The findings will enhance comprehension of COVID-19-associated AKI and inform targeted interventions for improved patient care. The coronavirus disease 2019 (COVID-19) pandemic has posed unprecedented challenges to global healthcare systems, with acute kidney injury (AKI) emerging as a significant complication among hospitalized patients. Although the prevalence and impact of COVID-19-associated AKI have been widely documented, the precise temporal trajectories of kidney function preceding and following this condition remain poorly understood. A comprehensive understanding of these trajectories is crucial for delineating the pathophysiological mechanisms underlying COVID-19-associated AKI and for devising effective management strategies. Despite the growing body of literature on COVID-19-associated AKI, there exists a critical research gap in the characterization of the temporal dynamics of kidney function in the context of this specific condition. Furthermore, while short-term outcomes of COVID-19-associated AKI have been investigated, there is a paucity of data on the long-term clinical sequelae, particularly focusing on mortality and impaired kidney function recovery post-hospitalization. In light of these gaps, this study aimed to comprehensively characterize the temporal trajectories of kidney function both preceding and following COVID-19-associated AKI in a large cohort of hospitalized patients. Additionally, we aimed to identify and analyze the clinical factors associated with mortality and impaired long-term kidney function recovery in this specific population. By addressing these objectives, we sought to provide valuable insights into the pathophysiology of COVID-19-associated AKI and contribute to the development of targeted interventions aimed at optimizing patient care and long-term outcomes in this vulnerable population. Prompt:* "Make the background more detailed"* Answer: The coronavirus disease 2019 (COVID-19) pandemic has triggered a global health crisis, profoundly affecting healthcare systems worldwide. Among the multifaceted complications associated with COVID-19, acute kidney injury (AKI) has emerged as a significant concern, contributing to the complexity of patient management and outcomes. Various studies have highlighted the prevalence and impact of COVID-19-associated AKI, revealing its association with heightened morbidity, prolonged hospital stays, and increased mortality rates. However, despite the growing awareness of this complication, the detailed temporal dynamics of kidney function before and after the onset of COVID-19-associated AKI have not been fully elucidated. A thorough understanding of the temporal trajectories of kidney function in the context of COVID-19-associated AKI is imperative for comprehensively unraveling the underlying pathophysiological mechanisms governing this intricate interplay. The progression of kidney dysfunction in the lead-up to AKI, as well as the subsequent recovery or deterioration of renal function post-AKI, remain areas that require in-depth investigation. Gaining insight into these trajectories can shed light on the critical windows of vulnerability and potential avenues for early intervention and management. Furthermore, while existing literature has primarily focused on short-term outcomes, there exists a notable research gap regarding the long-term clinical sequelae of COVID-19-associated AKI. Understanding the factors influencing both mortality rates and impaired long-term kidney function recovery among COVID-19 patients who have experienced AKI is essential for establishing comprehensive prognostic indicators and refining patient care strategies. Identifying these determinants can aid in risk stratification and facilitate the development of tailored interventions aimed at optimizing long-term renal health and overall patient outcomes. Given these critical gaps in the current knowledge base, the primary objective of this study was to conduct a comprehensive characterization of the temporal trajectories of kidney function preceding and following COVID-19-associated AKI in a substantial cohort of hospitalized patients. In tandem, the study sought to discern the clinical factors intricately linked with both mortality and impaired long-term kidney function recovery in this specific patient population. The findings of this study are anticipated to provide crucial insights into the pathophysiology of COVID-19-associated AKI and offer a foundation for the development of targeted interventions to enhance patient care and optimize long-term outcomes in this vulnerable patient cohort.

Supplementary file 2 (questionnaire)

Supplementary file 3 (questionnaire)

Supplementary file 4 (questionnaire)
